# Poster Session II - A249 PRECLINICAL CROHN’S DISEASE IS MARKED BY YEAST EXPANSION AND LOSS OF FILAMENTOUS FUNGI

**DOI:** 10.1093/jcag/gwaf042.249

**Published:** 2026-02-13

**Authors:** B Bharali, Q Li, A Griffiths, R Panaccione, L A Dieleman, H Steinhart, K Jacobson, K Croitoru, S Lee, W Turpin

**Affiliations:** Lunenfeld-Tanenbaum Research Institute, Toronto, ON, Canada; Lunenfeld-Tanenbaum Research Institute, Toronto, ON, Canada; The Hospital for Sick Children, Toronto, ON, Canada; University of Calgary, Calgary, AB, Canada; University of Alberta, Edmonton, AB, Canada; Lunenfeld Tanenbaum Research Institution, Sinai Health, Toronto, ON, Canada; BC Children’s Hospital, Vancouver, BC, Canada; Lunenfeld Tanenbaum Research Institution, Sinai Health, Toronto, ON, Canada; Lunenfeld Tanenbaum Research Institution, Sinai Health, Toronto, ON, Canada; Lunenfeld-Tanenbaum Research Institute, Toronto, ON, Canada

## Abstract

**Background:**

Alterations in the bacterial microbiome that precede Crohn’s disease (CD) have been described in prospective cohorts, but comparable data on gut fungi are limited. Patients with established CD, report expansion of Candida and Malassezia, reduced fungal diversity, and immune responses to fungal glycans. Genetic associations in fungal-sensing pathways (e.g., CARD9, Dectin-1) further support a possible role for fungi in IBD pathogenesis. Despite these implications, the contribution of the gut mycobiome to the earliest, preclinical phase of CD remains unknown.

**Aims:**

To establish whether specific fungal features are associated with risk of CD.

**Methods:**

A nested case-control subgroup from the CCC-GEM cohort was used for this study. Deep shotgun sequencing was done using baseline stool DNA. Reads were assembled with SPAdes, open reading frames predicted with Prodigal, and annotated by DIAMOND and MEGAN against NCBI nr. Fungal species-level abundances and functional profiles were tested with conditional logistic regression stratified by nested groups, yielding odds ratios (ORs) for future development of CD with multiple-testing correction with a significance of < 0.1.

**Results:**

The study population included 66 incident CD cases and 285 matched controls, with a median time to diagnosis of 2.5 years after sampling. The median age at baseline was 17 years, and 59% of participants were female. Several significant alterations in fungal species were evident years before diagnosis. Among those who went on to develop CD, filamentous fungi were depleted, including Laccaria bicolor (OR = 0.6, q = 0.005), Fonsecaea nubica (OR = 0.6, q = 0.005), and Dacryopinax primogenitus (OR = 0.7, q = 0.02). In contrast, Malassezia sp. (OR = 1.5, q = 0.1), Candida sojae (OR = 1.2, q < 0.1), and Blastomyces silverae (OR = 1.3, q = 0.009) were enriched in preCD. Functionally, enzymes such as chitin deacetylase (OR = 0.6, q0.005), cellulase (OR = 0.7, q = 0.02), and xyloglucanase (OR = 0.6, q0.01) were reduced in pre-CD, while prolyl oligopeptidase (OR = 1.3, q = 0.01), hydroxymethylglutaryl-CoA synthase (OR = 1.4, q = 0.005), and catalase (OR = 1.3, q = 0.04) were enriched.

**Conclusions:**

The pre-CD phase is marked by an association with reduced ecologically diverse filamentous fungi and enrichment of opportunistic yeast. Notably filamentous fungi, which are typically associated with ecological stability and plant polysaccharide metabolism, were significantly reduced. Functionally, a shift corresponding to a loss of fungal carbohydrate-degrading capacity, reflected by depleted enzymes involved in processing structural polysaccharides was observed. These alterations, detectable years before CD onset, suggest that environmental fungal differences may contribute to the earliest phases of CD pathogenesis.

**Funding Agencies:**

CCC, CIHR

